# Efforts for Construction of More Effective Prevention and More Inclusive Protection Systems in Matsudo City

**DOI:** 10.1093/geroni/igaa057.2379

**Published:** 2020-12-16

**Authors:** Tadashi Wada, Hitoshi Suda, Kana Sato

**Affiliations:** 1 Irahara Primary Care Hospital, Matsudo City, Chiba, Japan; 2 Seitoku University, Matsudo, Chiba, Japan; 3 Teikyo University of Science, Adachi, Tokyo, Japan

## Abstract

Matsudo City is preparing to implement an ordinance so that 3 protective services for children, older persons, and people with disabilities can work together for efficient prevention. Supporting pregnant women is sometimes effective for prevention of future child abuse. Supporting people with disabilities is often prevention of future neglect caused by them. Supporting perpetrators and victims of domestic violence is effective to prevent future elder abuse by interruption of abusive behaviors. We expect to prevent abuses by affiliation of various protective and supportive services. According to the Elder Abuse Prevention Law, elder abuse is defined as those inflicted by caregivers. Therefore, there is a big problem that abuses by non-caregivers are excluded from the coverage of the law. We are currently investigating abuses by non-caregivers to clarify the proportion of excluded cases among those reported to Elder Protective Services. I would like to talk about preliminary result of our investigation.

